# A method for my role as Executive Director: Gather, Empower and Deliver as one

**DOI:** 10.1093/eurpub/ckad197

**Published:** 2023-12-09

**Authors:** Charlotte Marchandise, Hans Henri P Kluge, Ricardo Mexia, Floris Barnhoorn

**Affiliations:** Executive Director EUPHA; Regional Director for WHO Europe; Chair of the 17th EPH Conference, Lisbon 2024; EPH Conference Office

##  

Dear readers,

I am deeply honoured to join the European Public Health Association (EUPHA). As I step into this role, I am inspired by collaborative spirit of EUPHA, and I am excited about the opportunities that lie ahead.

As I embark on my journey, I am genuinely impressed. Marieke Verschuuren’s column in October reminded us of the achievements over the past year.^1^ EUPHA’s ability to accomplish so much with its small secretariat and vast network of experts across Europe is truly commendable. Imagine what we can do if we manage to strengthen the team!

My approach to this role is one of humility and eagerness to learn, and recognizing the importance of continuity, and I am committed to building upon the legacy of EUPHA. At the same time, I am driven by a sense of optimism and a desire for innovation. As we navigate the challenges of our time, I believe that there is always room for growth and new aspirations. It is with this enthusiasm that I step in, ready to embrace change and explore new avenues for advancing public health in Europe and amplify your voices, our voices.

And this is urgent, as we are confronted with significant challenges.

At the time of writing, as we are preparing for the 16th European Public Health conference, scheduled for 8–11 November in Dublin, Ireland, our thoughts and concerns go to our colleagues from Israel’s Association of Public Health Physicians in the wake of the devastating terrorist attacks and hostage-taking in October. These tragic events have triggered a health and humanitarian crisis, resulting in tragic civilian deaths. Our thoughts also go out to the families and the health workers facing threats and the bombing in Gaza.

This gathering of public health professionals presents an opportunity to collectively advocate for the protection of people and the defence of human rights, inspired by the way colleagues have tirelessly worked on promoting peace and collaboration with their Palestinian counterparts over the past decades, and supporting them to the best of our ability. As WHO Director General Dr. Tedros Adhanom Ghebreyesus said: ‘In our fractured and divided world, we must continue to seek common ground and common good’. This is a matter of public health, moreover, this is what Public Health is about.

We are also facing the urgent need to address climate change’s adverse effects on public health. The consequences of climate change are not widely understood by the public or stakeholders, making it imperative for EUPHA to play a role in raising awareness and advocating for sustainable, health in all and for all policies.

For my first column here, I would like to share with you all the method I proposed while being interviewed for the position:

## First, Gather

We certainly know how to gather amongst Public Health actors, and this is our first role.

Yet, health is a political choice requiring champions. To amplify our voices within EUPHA and across other European organizations, we must strengthen our partnerships, and build strong relationships with institutions and political stakeholders at various levels. By engaging policymakers and translating evidence into policy, we can ensure that public health remains a priority on legislative agendas.

Collaboration with grassroots organizations and patients’ associations is equally vital. Their input is crucial in shaping public health policies that are inclusive and responsive to the diverse needs of our communities. Furthermore, we can broaden our collaborations with other sectors, such as cities, urban planners and architects, to create healthier and more sustainable living environments for European citizens.

## Then, Empower

Public health challenges are multifaceted, and EUPHA’s strength lies in promoting multidisciplinary research. Embracing the concept of boundary-spanning research will enable us to transcend traditional silos and address complex issues more effectively. Engaging citizens in research and decision-making processes can empower communities and foster active participation in public health initiatives.

EUPHA has made significant strides in training and mentoring through initiatives like EUPHAnxt. I believe we can do even better by utilizing design, infographics and social media to disseminate evidence effectively. Hosting regular webinars and knowledge-sharing sessions can enhance the exchange of scientific insights. EUPHA can play a pivotal role in educating and empowering influencers, from mayors to parliamentarians, to understand the importance of evidence-based public health policies.

And empowerment also relied on proper funding. Diversifying income sources is essential for ensuring financial sustainability.

## Finally, Deliver as one

Effective communication is at the heart of driving change—otherwise, health-adverse lobbies would not spend so much on commercials. EUPHA is recognized for publishing high-quality research, and we must continue to promote clear communication of findings to inform policymaking and public discourse. Expanding our coordinated advocacy efforts to engage with other sectors, such as education, transportation and economics, will amplify the impact of our message.

As a conclusion, while advocating for better global health policies for years, I embrace a guiding principle: ‘nothing for us, without us’. Therefore, above all else, I am eager to listen and learn from each of you. I invite you to reach out to me to engage in a meaningful dialogue, that will shape our upcoming work and the renewal of EUPHA’s strategy.

I am genuinely excited about the journey ahead and look forward to meeting you in Dublin and elsewhere.

Sincerely,

Charlotte Marchandise

## The one prerequisite for transforming health systems across our region: Trust

KlugeHans Henri PRegional Director for WHO Europe

In its absence, health workers are more likely to leave the profession, politicians are less willing to support health budgets and investments, and people like you and me, are at risk of poorer health outcomes or even completely avoidable deaths.

‘It’ refers to ‘trust’; trust in health systems, to be more precise.

Trust cannot be bought. It is fragile and can only be earned over time.

Loss or lack of trust is among the key factors hindering the achievement of healthy and prosperous societies and economies for all.

Loss of trust in health systems in the 53 member states of the WHO European Region is, as a matter of fact, among the greatest challenges we face in terms of public health.

Confronting unprecedented challenges—among them a pandemic, earthquakes, polio outbreaks, measles, mpox, accelerating climate change and armed conflict uprooting millions of people—the Region’s health systems have responded in hugely different ways. Some have succeeded in turning weaknesses into strengths, whereas others have struggled with overcoming the many hurdles on their path, fuelling further distrust.

The challenge here is 3-fold.

Firstly, people feel they can’t rely on the health system that ought to protect them when needed. They know that hospitals are understaffed, under-funded. They are aware of the long back-logs. On a greater scale, this distrust threatens the very solidarity that underpins the European social model. Social media, in this context, is both a culprit and part of the solution in this age of disinformation and misinformation where there is no scarcity of unverified health information, which too rarely is counteracted with scientific facts from trusted health authorities.

Secondly, more and more politicians doubt today’s health systems can meet changing health needs and are reluctant to increase health budgets for systems they see as outdated, incapable of change.

Thirdly, the very health workers, the backbone of health systems, fatigued as our Region emerges from COVID-19, don’t have faith in their working conditions changing for the better, their salaries being raised or their contribution to society being valued for what it is.

None of these challenges are new—they’ve simply been left unaddressed, festering amid the travails of the pandemic, and now erupting in the form of burnout, attrition and strikes.

Responding, on one hand, is relatively simple.

What an effective response requires, is strategic foresight and strong political will, to help ensure that we as a Region deliver on what we’ve committed to, to the 1 billion people we serve. That we act on the fact, after the many COVID-19 lessons for which we have paid dearly, that spending on health is not a cost but an investment.

Let’s untangle all of this, one response at a time.

To build public trust, health systems need to be revitalized. They need to enter the era of digital health to help address growing patient dissatisfaction with inefficient processes and long waiting times. Out-of-pocket payments for health need to be reduced under the umbrella of universal health coverage, and people need to feel that talk about UHC isn’t just empty words.

To gain the trust of politicians and attract sustained funds, health systems need to prove they are capable of transforming. Investment in and modernization of the health sector need to go hand in hand.

And finally, to regain health and care workers’ trust, they need to be acknowledged, educated, paid, valued and trained to embrace the rapid changes that innovation brings, if health systems are truly fit-for-purpose to avail of these opportunities.

The bottom line is that health systems—and remember that systems are created and delivered by people—need to show they are trustworthy. The concrete case for investment needs to be in place for policymakers to back.

In December this year, health workers and ministers, academics, politicians, together with experts from the WHO and the European Observatory on Health Systems and Policies, will be meeting in Tallinn, Estonia, to discuss trust and the transformation ahead—with people at the very centre.

Because systems do not transform themselves. People transform systems, and that can only be done in a climate of trust and co-creation.

The principles underlying Tallinn 2023—building on the groundbreaking Tallin Charter of 2008—are from the European Programme of Work, 2020–5 and its vision for a profound transformation of health systems and social care in the Region, with the vision of a digital, innovative, outcomes-directed and people-centred future.

The Charter was the product of a very different era. Our current day and age is marked by geopolitics more fluid than ever, galloping inflation and cost-of-living indices, extreme weather events triggering ever-mounting calamities, an infodemic marring social interactions. And of course, we are emerging from a pandemic labelled the biggest global crisis since World War Two.

Yet, our current day and age is also one of unprecedented opportunity.

Scientific and medical advances we—let alone the previous generation—could hardly have foreseen even a few years ago. IT and digital health marvels that have the potential to transform health and well-being. A world in which youth constitute one out of every three persons—a demographic reality that can be harnessed for a better future, if we allow it.

Amid our present reality, if we are to reform and make our health systems resilient enough to meet future health threats; if we are to galvanize regional support for the values espoused back in 2008, reinstating trust is the prerequisite.

## Sailing the waves of European public health: exploring a sea of innovation

MexiaRicardoChair of the 17th EPH Conference, Lisbon 2024BarnhoornFlorisEPH Conference Office

17th European Public Health Conference

12–15 November 2024

Lisbon Conference Centre (LCC), Lisbon, Portugal

WELCOME TO LISBON 2024

The theme of next year’s EPH Conference ‘Sailing the waves of European public health: exploring a sea of innovation’ captures our collective mission in public health. Just as explorers once set sail to discover new worlds, we, as public health professionals, embark on a journey of innovation and discovery in our field. We seek to push the boundaries, discover new approaches and chart the course for the future of public health in Europe.

In five plenary sessions we will discuss current questions and challenges that we will face within the public health field in the coming years.

1. Artificial Intelligence in Public Health: Lisbon, a city that bridges tradition and modernity, is the ideal setting to delve into the cutting-edge realm of Artificial Intelligence (AI) in public health. We’ll explore how AI is transforming disease surveillance, healthcare delivery and decision-making, propelling us into an exciting era of health innovation.
2. One Health—Animal Approach: Lisbon’s maritime heritage resonates with the One Health concept, which recognizes the interconnectedness of human, animal and environmental health. Against the backdrop of our historic port, we’ll discuss how taking an animal-centric approach can strengthen public health strategies and mitigate emerging threats.
3. Social Marketing in Public Health: Lisbon’s vibrant street life and dynamic culture provide a unique framework to discuss the art of social marketing in public health. Join us in exploring innovative campaigns, digital strategies, and community engagement techniques to promote healthier behaviours.
4. Health Inequalities: The diverse neighbourhoods of Lisbon, each with its own character and challenges, serve as a reminder of the widespread health inequalities in Europe that persist across the continent. There will be a discussion on innovative strategies that can be used to reduce disparities and ensure equitable access to health care.
5. Global Health: Lisbon’s status as a global port city reminds us of the interconnectedness of our world in an increasingly globalized world. Together, we’ll discuss how European public health efforts can contribute to global health solutions, addressing issues that transcend borders.

The plenary programme is organized by EPH Conference Foundation, European Public Health Association (EUPHA), EuroHealthNet, European Health Management Association (EHMA). ASPHER, European Observatory on Health Systems and Policies, European Centre for Disease Prevention and Control (ECDC), European Commission and WHO Regional Office for Europe.

JOIN US!

As the host city for the 17th European Public Health Conference, Lisbon invites you to embark on a journey of exploration. Nestled along the captivating shores of the Atlantic Ocean, Lisbon is a city where history, culture, and innovation converge. With its charming streets, historic neighbourhoods, and a vibrant arts scene, Lisbon offers an ideal location for next year’s conference.

In the heart of this beautiful city, the EPH Conference in Lisbon 2024 promises to be an unforgettable experience. Whether you’re strolling through historic Alfama addressing the challenges of global health, discussing AI innovations while tasting a delicious ‘pastel de Belém’, or debating strategies to combat health inequalities overlooking the Tagus River, you’ll find inspiration in every corner of Lisbon.

Set your calendars for this event and prepare yourself to embark on a voyage of innovation in the field of public health. Lisbon awaits your arrival, ready to welcome you to a conference that will surprise you and leave a lasting impression.

See you in Lisbon!
